# Urban COVID-19 endemism in Petrópolis: detection of an endemic focus by spatial analysis

**DOI:** 10.17843/rpmesp.2023.402.11341

**Published:** 2023-06-30

**Authors:** Felix J. Rosenberg, Caiett Genial, Bruno Cesar dos Santos

**Affiliations:** 1 Fórum Itaboraí, Fiocruz, Petrópolis, Brasil. Fórum Itaboraí Fiocruz, Petrópolis Brasil

**Keywords:** SARS-CoV-2, COVID-19, Pandemics, Ecoepidemiology, Disease Hotspot, Spatial Analysis, Brazil

## Abstract

This study aimed to identify an ecosystem of urban endemism that explains the persistence of SARS-CoV-2 during the first 18 months of the pandemic in the municipality of Petrópolis, Rio de Janeiro, Brazil. We analyzed official records of monthly COVID-19 cases, georeferenced according to the residence address of each confirmed case. Monthly heat maps identifying points with different spatial densities of the disease were constructed by applying the kernel methodology. Heat spots with five intensity levels were identified for the spatial density of cases. The points of highest intensity, known as hotspots, remained constant throughout the period in a polygon of approximately 4 km^2^ located in the center of the city of Petrópolis. In conclusion, we found that the highest concentration of cases remained in the same location over time, despite the sporadic dispersion of cases within the municipal territory.

## INTRODUCTION

During the COVID-19 pandemic, several researchers used spatial analysis methods to identify the territorial determinants of its distribution, focusing on social inequalities and inequities in access to medical care. These studies have covered the risk of the disease at the regional or municipal level, using statistical records from the epidemiological surveillance systems of different countries, such as Ecuador ^1^, Mexico ^2^, Portugal ^3^, Germany ^4^, among others. In addition, there are extensive literature reviews on the use of geographic information analysis methods, particularly in China ^5^ and the United States ^6^.

Some authors have used heat maps to identify areas with a high relative density of cases, known as hotspots, in places as diverse as the state of Sergipe in Brazil ^7^ and Punjab in Pakistan ^8^. These studies have analyzed epidemiological surveillance data according to geopolitical divisions or through the use of heat maps of different scales, in order to define and describe areas of higher or lower epidemic risk at different times of the pandemic. In all cases, the virus is assumed to have been introduced in areas with a high risk of exposure, and it is considered that these hotspots experience epidemic outbreaks of different magnitudes depending on the size and density of the territory, but that they tend to decrease when the immunity of the exposed population reaches a certain level.

Over the last century, several concepts have been constructed on the basis of the “natural nidality of communicable diseases” developed by Pavlovsky ^9^; these concepts highlight the relevance of geographic space in determining the health-disease process ^10^. This allows us to analyze, in an integrated and dialectical manner, the effects of human intervention on environmental and climatic changes, as well as their impact on health, particularly regarding the emergence of new communicable diseases ^11^. In this context, the theory of the natural niches of communicable diseases was developed, which includes proposals for the control and even eradication of causal agents, in this respect Roche states that “we can define the niche of an organism as the position of this organism within its environment, available resources and competitors. Then, it is characterized by all the conditions required to sustain a viable population of the organism, in space and time.” ^12^.

Normally, these perspectives are applied to rural spaces or large geographical areas where different elements related with the viability, maintenance and transmission of the infectious agent coexist, including vectors or reservoirs, in addition to the susceptible host ^13^. This is considered an endemic condition, that is, a geographic space in which the infection is constantly maintained at a level that allows the continuous transmission of the disease ^13^. Thus, this study aimed to identify an ecosystem of urban endemism that explains the persistence of SARS-CoV-2 during the first 18 months of the pandemic in the municipality of Petrópolis, Rio de Janeiro, Brazil.

**KEY MESSAGES**

Motivation for the study. During the COVID-19 pandemic, spatial analysis methodologies were used to identify the territorial determinants of its distribution and social inequalities in access to medical care.

Main findings. We found a concentration of cases in a specific location of the municipality, which remained constant throughout the study period, with sporadic outbreaks in other areas.

Implications. It is necessary to pay attention to possible endemic foci of viral diseases in urban settings and to take measures to eliminate them as well as to prevent their spread within and outside the area.

## THE STUDY

We carried out a descriptive and spatial analysis of confirmed cases of SARS-CoV-2 infection in the municipality of Petrópolis, located in the State of Rio de Janeiro, at a distance of approximately 70 km from the city of Rio de Janeiro. The municipality has a total area of 795,798 km^2^ and an average altitude of 738 m. According to projections, the total population was 306,678 inhabitants in 2020.

A total of 49,050 records of confirmed SARS-CoV-2 cases during the period from March 1, 2020 to August 31, 2021 (Table 1) were geocoded. The data were provided by the Municipal Health Secretariat - SMS/Petrópolis. We used the QuantumGIS® software to produce the maps and the Google® cartographic base was used for geocoding (Google Maps® API). In cases where the algorithm presented errors, such as in unnumbered routes, passages, avenues or lanes, data was verified by analyzing satellite images from Google Earth® and on-site visits to the identified addresses. In order to guarantee anonymity of the records, monthly heat maps were prepared that identified clusters with a higher spatial density of cases.

**Table 1:** Number of monthly records of geocoded COVID-19 cases in the Municipality of Petrópolis (March 2020 to August 2021).

Month and year	n
March 2020	41
April 2020	795
May 2020	1012
June 2020	1127
July 2020	1129
August 2020	1044
September 2020	1065
October 2020	578
November 2020	1173
December 2020	2949
January 2021	2677
February 2021	1381
March 2021	2319
April 2021	2945
May 2021	7918
June 2021	6710
July 2021	5737
August 2021	8428

Source: Prepared by the authors based on data from the Municipal Health Secretariat of Petrópolis.

The kernel density estimation, widely known and used in the epidemiological context ^14^, was used to identify the areas with the highest concentration of cases. This estimation transforms the vector layer of points (georeferenced records) into a monochromatic raster data showing the concentration of cases across 30 cm pixels. The kernel density is a function that counts all points within a given region of influence, with probabilistic weighting based on the distance of each point from the location of interest ^15^^-^^17^.

We used two basic parameters for applying the kernel density method: the radius of influence and the k function ^18^. The radius of influence defines the area centered on the estimation point, which indicates how many events contribute to the estimate of the intensity function λ ^15^. For this study, we determined a radius of influence of 1 km, based on previous research ^18^.

The k function is less relevant than the radius of influence, since an alteration of the radius affects the entire possible interpolation field ^19^, which is more critical for the representation of the phenomena. We chose the quartic function among the most commonly used functions, which proved to be the most effective one in identifying clusters of cases (hotspots) within the chosen radius, without losing contact areas between hotspots (average areas), which made it possible to analyze the expansion or retraction of the virus over time, as well as its persistence in specific areas.

In order to better identify the physical area of the hotspots with the highest intensity and to monitor monthly disease persistence, a coordinate grid was created with squares of 1 km^2^ area, using the equirectangular projected coordinate system, in a layout similar to a Cartesian plane ^20^. Maps generated by kernel interpolation were overlaid on the squares to calculate a persistence score for each km^2^. The areas of highest heat intensity were transformed into vector data, where each pixel produced by the kernel interpolator was converted into a point with a weight proportional to its density. In this way, approximately 11 million pixel points were obtained for each 1 km square. The vector values of each of the monthly maps produced by kernel interpolation were summed, generating a quantifiable value for each square. These values were classified using the “false color” representation, simulating a multiband raster, which allowed the creation of a taxonomy of three-color spectra: blue (minimum values), yellow (intermediate values) and red (maximum values), with a gradient of five classes, including two intermediate classes.

Data from the 2010 Census ^21^, corresponding to the sectors relevant to the study and projected to the total population estimated for 2021 ^22^, were used to characterize the area with the highest concentration of cases. Other relevant comments about the area were obtained through face-to-face visits by the researchers.

The Municipal Health Secretariat of the Municipality of Petrópolis authorized and provided the household records of the confirmed cases of COVID-19 as part of its epidemiological surveillance actions.

## FINDINGS

**Figure 1:**
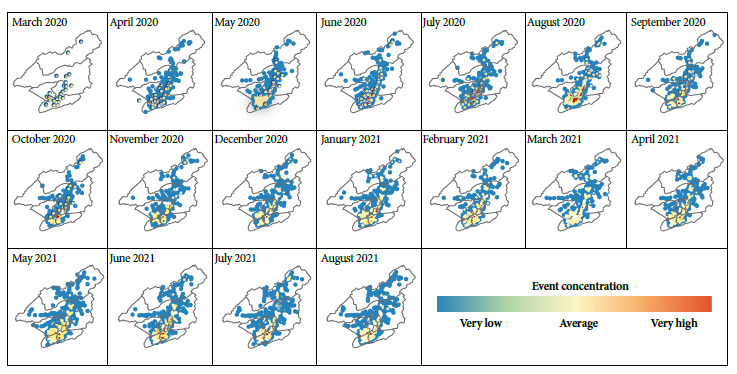
Monthly maps of COVID-19 records in the municipality of Petrópolis based on the kernel density methodology (March 2020 to August 2021).

We found that, from April 2020 onwards, the same location had the highest concentration of cases for the following 18 months, regardless of the total number of records in each month and the dispersion of cases in the municipal territory (Figure 1).

**Figure 2:**
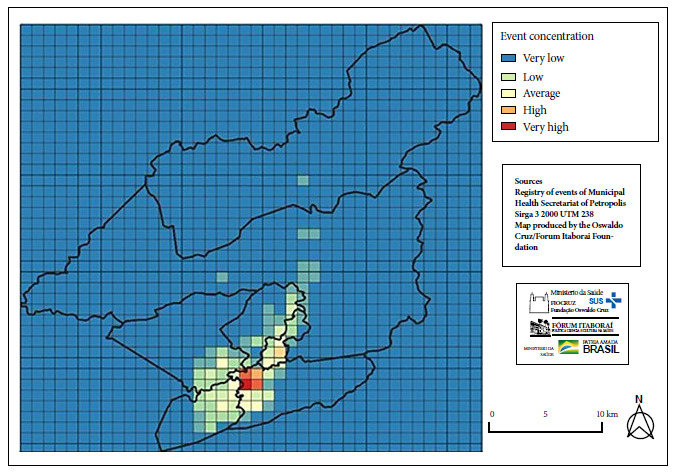
Areas of higher persistence of COVID-19 cases through the determination of vector values of 1 km² quadrants (March 2020 to August 2021).

Four quadrants with higher persistence were identified by adding the vector values corresponding to each month in each square of the 1 km^2^ coordinate system (Figure 2), forming a polygon similar to the areas of higher heat intensity observed monthly. Therefore, we identified that the epidemic in Petrópolis was concentrated in a single location, with sporadic outbreaks in other quadrants of the municipality.

**Figure 3:**
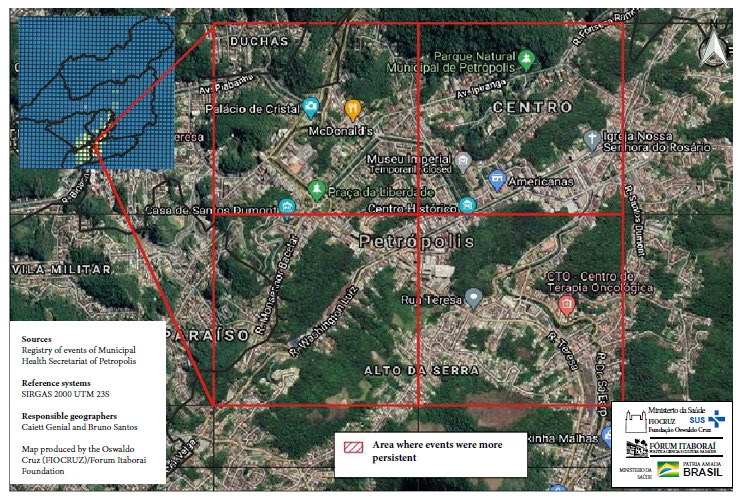
Area of higher persistence of COVID-19 in Petrópolis, enlarged with GoogleEarth® satellite images (March 2020 to August 2021).

To identify this area of persistence, the Google ® satellite photo was superimposed on the cartographic base of the selected grids, which allowed the identification of the area corresponding to the “Historic Center” of the city and its immediate surroundings (Figure 3).

Other central areas of the municipality also presented a considerable number of registered cases. However, this situation was not persistent throughout the period, suggesting the occurrence of possible localized outbreaks in time and space.

## DISCUSSION

The use of spatial epidemiology in our study allowed us to understand the population dynamics of the disease, based on the urban organization of the municipality and the sociodemographic conditions. This provides the possibility of designing specific strategies to address each of these conditions, taking into account not only individual risk factors and virus transmission during epidemics or outbreaks, but also the characteristics of disease persistence in a given territory.

Most epidemiological analyses of the COVID-19 pandemic assume a transmission cycle that begins with the introduction of the agent into a given territory, followed by community transmission and the subsequent epidemic spread with transmission rates (Tr) greater than 1. This cycle is characterized by an initial increase in cases, followed by a plateau with transmission rates near 1, and finally a decrease as the number of susceptible individuals decreases. These successive epidemic waves may be attributed to the relaxation of social isolation measures ^23^ or to the introduction of new variants of the virus.

These reasons explain the persistence of the pandemic for more than 18 months at the global or national level, until finally a consistent decline occurred thanks to the high vaccination coverage achieved in developed countries. Therefore, health services around the world have focused on control mechanisms through sanitary barriers, whether at the international, national or local level. However, these measures, which have been effective in containing pandemics and more localized epidemic outbreaks in the past, appear to have been insufficient to contain the current COVID-19 pandemic. One possible reason for the shortfall of this strategy may be the lack of attention paid to potential endemic foci, i.e., “natural nests of the virus” in urban settings.

In this study, we considered a radius of 1 km to outline the heat circles. Theoretically, in territories of this size, a hotspot should rapidly achieve enough herd immunity to stop or at least significantly reduce virus transmission ^24^. However, the largest hot spot in the entire municipal territory was consistent during 18 months since the beginning of the epidemic, with small shifts that always remained within a polygon of less than 4 km^2^. Identifying the mechanisms by which the SARS-CoV-2 virus remains endemic would allow the implementation of measures that could not only eliminate this hotspot, but also prevent the effects of transmission within and beyond this endemic area.

The identified hotspot contains demographic and urban elements that explain the phenomenon. Petrópolis is a highly centralized municipality, like many in Brazil ^25^. The hotspot area coincides with the downtown area, which contains approximately 7% of the population. The tallest buildings in the city are located there, as well as a precarious community of significant size. This community interacts constantly with the nodal center of the polygon, 1-2 km^2^ away. The center concentrates federal, state and municipal public services, mostly exclusive to the entire municipality. In addition, there are medical offices, supermarkets, offices, popular and exclusive stores, markets and street fairs. There are also museums and tourist attractions, which generate a constant flow of municipal public transportation to the area.

Our study has some limitations. First, we used data collected during a specific period (March 1, 2020 to August 31, 2021) in the municipality of Petrópolis, Rio de Janeiro, Brazil. This means that the findings and conclusions of the study are applicable only to that period and geographic location, limiting their applicability to other areas or times during the pandemic. In addition, the study focuses on the use of spatial analysis methodologies and heat maps to identify areas of high concentration of cases, known as hotspots. While this provides valuable information on the spatial distribution of COVID-19 in Petrópolis, it is important to keep in mind that heat maps can be sensitive to variations of the population and event density, which may result in bias. In addition, the study is based on data provided by the Municipal Health Secretariat of Petrópolis, which raises the possibility of underreporting or biases in the data collected. Therefore, the results should be interpreted carefully and further studies are needed to corroborate and extend these findings to other geographical and temporal contexts.

In conclusion, this study identified an urban endemic focus of COVID-19 in the municipality of Petrópolis, Rio de Janeiro, Brazil, during the first 18 months of the pandemic. Using spatial analysis, we found that the highest concentration of cases remained in the same location over time, despite the dispersion of cases throughout the municipal territory. The persistence of the endemic focus over time raises questions about the control strategies used so far, as they do not seem to have been sufficient to contain the spread of the virus in this specific area.
