# Supplementary material for: Urban COVID-19 endemism in Petrópolis: detection of an endemic focus by spatial analysis
Source: Rev Peru Med Exp Salud Publica. 2023 Jun 30;40(2):213–9. doi: 10.17843/rpmesp.2023.402.11341 (PMC10953638; doi:10.17843/rpmesp.2023.402.11341)
Supplement: Translation

# Translation: Endemismo urbano por COVID-19 en Petrópolis: Detección de un foco endémico mediante análisis espacial

**Authors:** Felix J. Rosenberg, Caiett Genial, Bruno Cesar dos Santos

**Affiliations:** 1 Fórum Itaboraí, Fiocruz, Petrópolis, Brasil.

**Keywords:** SARS-CoV-2, COVID-19, Pandemias, Ecoepidemiología, Punto Alto de Contagio de Enfermedades, Análisis Espacial, Brasil

## Abstract

Se realizó un estudio con el objetivo de identificar un ecosistema de endemismo urbano que explique la persistencia del SARS-CoV-2 durante los primeros 18 meses de la pandemia en el municipio de Petrópolis, Río de Janeiro, Brasil. Se analizaron los registros oficiales de casos mensuales de COVID-19, georreferenciados según el domicilio de residencia de cada caso confirmado y se elaboraron mapas de calor mensuales que identifican puntos con diferentes densidades espaciales de la enfermedad mediante la aplicación de la metodología de kernel. Se identificaron puntos de calor con cinco niveles de intensidad para la densidad espacial de casos. Los puntos de mayor intensidad, conocidos como «hotspots», se mantuvieron constantes durante todo el período en un polígono de aproximadamente 4 km^2^ ubicado en el centro de la ciudad de Petrópolis. En conclusión, se encontró que la mayor concentración de casos se mantuvo en la misma ubicación a lo largo del tiempo, a pesar de la dispersión esporádica de los casos en el territorio municipal.

## INTRODUCCIÓN

Durante la pandemia de COVID-19, varios investigadores utilizaron metodologías de análisis espacial para identificar los determinantes territoriales de su distribución, centrándose en las desigualdades sociales y las inequidades en el acceso a la atención médica. Estos estudios han abarcado el riesgo de la enfermedad a nivel regional o municipal, utilizando registros estadísticos de sistemas de vigilancia epidemiológica en diferentes países como Ecuador ^1^, México ^2^, Portugal ^3^, Alemania ^4^, entre otros. Además, se han realizado extensas revisiones bibliográficas sobre el uso de métodos de análisis de información geográfica, destacando trabajos realizados en China ^5^ y en Estados Unidos ^6^.

Algunos autores han utilizado mapas de calor para identificar áreas con una alta densidad relativa de casos, conocidas como «hotspots», en lugares tan diversos como el estado de Sergipe en Brasil ^7^ y Punjab en Pakistán ^8^. Estos estudios han analizado datos de vigilancia epidemiológica según divisiones geopolíticas o mediante el uso de mapas de calor de diferentes escalas, con el fin de definir y describir áreas de mayor o menor riesgo epidémico en diferentes momentos de la pandemia. En todos los casos, se parte de la hipótesis de que el virus se introduce en áreas con un alto riesgo de exposición, y se considera que estos «hotspots» experimentan brotes epidémicos de diferentes magnitudes dependiendo del tamaño y densidad del territorio involucrado, pero que tienden a disminuir cuando la inmunidad de la población expuesta alcanza cierto nivel.

A partir de la noción de «nidalidad natural de las enfermedades transmisibles» desarrollada por Pavlovsky ^9^ en el siglo pasado, fueron construidos varios conceptos que resaltan la relevancia del espacio geográfico en la determinación del proceso salud-enfermedad ^10^. Esto nos permite analizar de manera integrada y dialéctica los efectos de la intervención humana en los cambios ambientales y climáticos, y su impacto en la salud, particularmente en la aparición de nuevas enfermedades transmisibles ^11^. En este contexto, se desarrolla la teoría de los nichos naturales de las enfermedades transmisibles, que incluye propuestas de control e incluso erradicación de los agentes causales, al respecto Roche plantea que «un nicho de un organismo se refiere a su posición dentro de su entorno, sus recursos y sus competidores. Está caracterizado por todas las condiciones necesarias para mantener una población viable del organismo en el espacio y en el tiempo» ^12^.

Normalmente, estas perspectivas se aplican a espacios rurales o a grandes áreas geográficas donde coexisten diferentes elementos que intervienen en la viabilidad, el mantenimiento y la transmisión del agente infeccioso, incluyendo vectores o reservorios, además del huésped susceptible ^13^. Esto se considera una condición endémica, es decir, un espacio geográfico en el cual la infección se mantiene constantemente a un nivel que permite la transmisión continua de la enfermedad en su población ^13^. Así, el objetivo del presente estudio fue identificar un ecosistema de endemismo urbano que explique la persistencia del SARS-CoV-2 durante los primeros 18 meses de la pandemia en la municipalidad de Petrópolis, Río de Janeiro, Brasil.

**MENSAJE CLAVE**

Motivación para realizar el estudio. Durante la pandemia de COVID-19, se han utilizado metodologías de análisis espacial para identificar los determinantes territoriales de su distribución y las desigualdades sociales en el acceso a la atención médica.

Principales hallazgos. Se observó una concentración de casos en una ubicación específica del municipio, que se mantuvo constante a lo largo del periodo estudiado, con brotes esporádicos en otras áreas.

Implicancias. Es necesario prestar atención a posibles focos de endemia de las enfermedades virales en entornos urbanos y tomar medidas para eliminarlos y evitar su propagación dentro y fuera de ellos.

## EL ESTUDIO

Se realizó un análisis descriptivo y espacial de casos confirmados con SARS-CoV-2 en el Municipio de Petrópolis, ubicado en el Estado de Río de Janeiro, a una distancia aproximada de 70 km de la ciudad de Rio de Janeiro. El municipio tiene una superficie total de 795 798 km² y una altitud media de 738 m. Según las proyecciones para el año 2020, la población total era de 306 678 habitantes.

Se geocodificó un total de 49 050 registros de casos confirmados de SARS-CoV-2, durante el período del 1 de marzo de 2020 al 31 de agosto de 2021 (Tabla 1). Los datos utilizados fueron proporcionados por la Secretaría Municipal de Salud - SMS/Petrópolis. Para la geocodificación, se utilizó el software QuantumGIS**®** para la elaboración de los mapas y la base cartográfica de Google® para la geocodificación (API Google Maps®). En casos donde el algoritmo presentaba errores, como en rutas sin numeración, pasajes, avenidas o carriles, se realizó la verificación mediante el análisis de imágenes satelitales en Google Earth® y visitas *in situ* a los domicilios identificados. Con el fin de garantizar el anonimato de los registros, se elaboraron mapas mensuales de calor que identificaron agrupamientos (clusters) con mayor densidad espacial de casos.

**Tabla 1:** Número de registros mensuales de casos de COVID-19 geocodificados en el Municipio de Petrópolis (marzo de 2020 a agosto de 2021).

Mes y año	n
Marzo 2020	41
Abril 2020	795
Mayo 2020	1012
Junio 2020	1127
Julio 2020	1129
Agosto 2020	1044
Setiembre 2020	1065
Octubre 2020	578
Noviembre 2020	1173
Diciembre 2020	2949
Enero 2021	2677
Febrero 2021	1381
Marzo 2021	2319
Abril 2021	2945
Mayo 2021	7918
Junio 2021	6710
Julio 2021	5737
Agosto 2021	8428

Fuente: Elaborado por los autores a partir de datos de la Secretaría Municipal de Salud de Petrópolis.

Se utilizó la estimación de concentración de densidad «kernel», ampliamente conocida y utilizada en el contexto epidemiológico ^14^, para identificar las áreas de mayor concentración de casos. Esta estimación transforma la capa vectorial de puntos (registros georreferenciados) en un dato de tipo matricial (raster) monocromático que muestra la concentración de casos a través de píxeles de 30 cm. La densidad de kernel es una función que cuenta todos los puntos dentro de una región de influencia determinada, con una ponderación probabilística basada en la distancia de cada punto desde la ubicación de interés ^15^^-^^17^.

Para la aplicación de la densidad de kernel, se utilizaron dos parámetros básicos: el radio de influencia y la función k ^18^. El radio de influencia define el área centrada en el punto de estimación, que indica cuántos eventos contribuyen a la estimación de la función de intensidad λ ^15^. Para este estudio, se determinó un radio de influencia de 1 km, basado en investigaciones previas ^18^.

La función k tiene menor relevancia que el radio de influencia, ya que una alteración en el radio afecta todo el campo de interpolación posible ^19^, lo cual es más crítico para la representación de los fenómenos. Entre las funciones más utilizadas, se optó por la función cuártica, que se mostró más eficaz para identificar conglomerados de casos (*hotspots*) en el radio elegido, sin perder áreas de contacto entre los *hotspots* (áreas promedio), lo que permitió analizar la expansión o retracción del virus a lo largo del tiempo, así como su persistencia en áreas específicas.

Con el fin de identificar mejor el área física correspondiente a los *hotspots* de mayor intensidad y monitorear la persistencia mensual de la enfermedad, se creó un cuadriculado de coordenadas con cuadrados de 1 km² de área, utilizando el sistema de coordenadas proyectadas equirectangular, en una disposición similar a un plano cartesiano ^20^. Los mapas generados por la interpolación de kernel se superpusieron a los cuadrados para calcular un score de persistencia para cada km². Las áreas de mayor intensidad de calor se transformaron en datos vectoriales, donde cada píxel producido por el interpolador kernel se convirtió en un punto con un peso proporcional a su densidad. De esta manera, se obtuvieron aproximadamente 11 millones de puntos en forma de píxeles en cada cuadrado de 1 km. Los valores vectoriales de cada uno de los mapas mensuales producidos por la interpolación kernel se sumaron, generando un valor cuantificable para cada cuadrado. Estos valores se clasificaron mediante la representación de «color falso», simulando un raster multibanda, lo que permitió crear una taxonomía de tres espectros de color: azul (valores mínimos), amarillo (valores intermedios) y rojo (valores máximos), con una graduación de cinco clases, incluyendo dos clases intermedias.

Se utilizaron los datos del Censo de 2010 ^21^, correspondientes a los sectores censales pertinentes al área de estudio, proyectados a la población total estimada para 2021 ^22^, para caracterizar el área de mayor concentración de casos. Otras observaciones relevantes sobre el área se obtuvieron mediante visitas presenciales de los investigadores.

La Secretaría Municipal de Salud del Municipio de Petrópolis autorizó y proporcionó los registros domiciliares de los casos confirmados de COVID-19 como parte de sus acciones de vigilancia epidemiológica.

## HALLAZGOS

A partir del mes de abril de 2020, se observa que la mayor concentración de casos se mantiene en la misma ubicación durante los 18 meses siguientes, independientemente del número total de registros en cada mes y de la dispersión de los casos en el territorio municipal (Figura 1).

**Figura 1:** Mapas mensuales de registros de COVID-19 en el municipio de Petrópolis a partir de la metodología de densidad kernel (marzo de 2020 a agosto de 2021).

Al sumar los valores vectoriales correspondientes a cada mes en cada cuadrado del sistema de coordenadas de 1 km², se identifican cuatro cuadrantes con una persistencia mayor (Figura 2), formando un polígono similar a las áreas de mayor intensidad de calor observadas mensualmente. Identificando que la epidemia en Petrópolis se concentra en una única ubicación, con brotes esporádicos en otros cuadrantes del municipio.

**Figura 2:** Áreas de mayor persistencia de casos de COVID-19 a través de la determinación de valores vectoriales de cuadrantes de 1 km² (marzo de 2020 a agosto de 2021).

Para identificar esta área de persistencia, se superpuso la foto satelital de Google® a la base cartográfica de las cuadrículas seleccionadas, lo que permitió identificar el área correspondiente al «Centro Histórico» de la ciudad y su entorno inmediato (Figura 3).

**Figura 3:** Área de mayor persistencia de COVID-19 en Petrópolis, ampliada con imagen de satélite a través de GoogleEarth® (marzo de 2020 a agosto de 2021).

Otras áreas centrales del municipio también presentaron un nivel considerable de casos registrados. Sin embargo, esta situación no fue persistente a lo largo del periodo, lo que sugiere la ocurrencia de posibles brotes localizados en el tiempo y el espacio.

## DISCUSIÓN

En el presente estudio, el uso de la epidemiología espacial permite comprender la dinámica poblacional de la enfermedad, basándose en la organización urbanística del municipio y las correspondientes realidades sociodemográficas. Esto brinda la posibilidad de diseñar estrategias específicas para abordar cada una de estas realidades, teniendo en cuenta no solo los factores de riesgo individual y la transmisión del virus durante momentos epidémicos o brotes agudos, sino también las características de persistencia de la enfermedad en un territorio dado.

En la mayoría de los análisis epidemiológicos de la pandemia de COVID-19, se parte de la premisa de un ciclo de transmisión que comienza con la introducción del agente en un territorio determinado, seguido de la transmisión comunitaria y la consecuente propagación epidémica con tasas de transmisión (Tr) superiores a 1. Este ciclo se caracteriza por un aumento inicial de casos, seguido de una meseta en las tasas de transmisión cercanas a 1, y finalmente una disminución a medida que los individuos susceptibles se vuelven menos numerosos en el territorio. Estas sucesivas olas epidémicas pueden ser atribuidas al relajamiento de las medidas de aislamiento social ^23^ o a la introducción de nuevas variantes del virus.

Estas explicaciones justifican la persistencia de la pandemia durante más de 18 meses a nivel global o nacional, hasta que finalmente se observa una disminución consistente gracias a las altas coberturas de vacunación alcanzadas en países desarrollados. Por lo tanto, los servicios de salud en todo el mundo se han enfocado en los mecanismos de control a través de barreras sanitarias, ya sea a nivel internacional, nacional o local. Sin embargo, estas medidas, que han sido efectivas en la contención de pandemias globales y brotes epidémicos más localizados en el pasado, parecen no haber sido suficientes para contener la actual pandemia de COVID-19. Una posible razón que contribuye a explicar la insuficiencia de esta estrategia puede ser la falta de atención prestada a posibles focos de endemia del virus, es decir, «nidos naturales del virus» en entornos urbanos.

En este estudio, se utilizó un radio de 1 km para delinear los círculos de calor. Teóricamente, en territorios de esta dimensión, un foco de calor debería alcanzar rápidamente una inmunidad de rebaño suficiente para extinguir o al menos reducir de manera significativa la transmisión del virus ^24^. Sin embargo, se observó que la mayor mancha de calor en todo el territorio municipal se registró de manera constante durante 18 periodos mensuales desde el inicio de la epidemia, con pequeños desplazamientos que siempre se mantuvieron dentro de un polígono de menos de 4 km². Identificar los mecanismos por los cuales el virus SARS-CoV-2 se mantiene endémico permitiría implementar medidas que no solo eliminaran este foco, sino que también evitaran los efectos de esta área endémica en la transmisión dentro y fuera de ella.

El foco de calor identificado concentra elementos demográficos y urbanísticos que explican el fenómeno. Petrópolis es una municipalidad altamente centralizada, como muchas en Brasil ^25^. El área de calor coincide con la zona céntrica, albergando aproximadamente el 7% de la población. Allí se encuentran los edificios más altos y una comunidad precarizada de tamaño significativo. Esta comunidad interactúa constantemente con el centro nodal del polígono, a 1-2 km² de distancia. El centro concentra servicios públicos federales, estatales y municipales, mayormente exclusivos para todo el municipio. Además, hay consultorios médicos, oficinas, supermercados, tiendas populares y exclusivas, mercados y ferias callejeras. También hay museos y atracciones turísticas, generando un flujo constante de transporte público municipal hacia el área.

El presente estudio tiene algunas limitaciones que deben tenerse en cuenta. En primer lugar, se basa en datos recopilados durante un período específico (del 1 de marzo de 2020 al 31 de agosto de 2021) en la municipalidad de Petrópolis, Río de Janeiro, Brasil. Esto significa que los hallazgos y conclusiones del estudio son aplicables solo a ese período y ubicación geográfica, lo que limita su generalización a otras áreas o momentos de la pandemia. Además, el estudio se centra en el uso de metodologías de análisis espacial y mapas de calor para identificar áreas de alta concentración de casos, conocidas como «hotspots». Si bien esto proporciona información valiosa sobre la distribución espacial del COVID-19 en Petrópolis, es importante tener en cuenta que los mapas de calor pueden ser sensibles a las variaciones en la densidad de población y eventos, lo que puede sesgar los resultados. Además, el estudio se basa en datos proporcionados por la Secretaría Municipal de Salud de Petrópolis, lo que plantea la posibilidad de que haya subregistro o sesgos en los datos recopilados. Por lo tanto, se requiere precaución al interpretar los resultados y se necesitan estudios adicionales para corroborar y ampliar estos hallazgos en otros contextos geográficos y temporales.

En conclusión, este estudio identificó un foco endémico urbano de COVID-19 en la municipalidad de Petrópolis, Río de Janeiro, Brasil, durante los primeros 18 meses de la pandemia. Utilizando un análisis espacial, se encontró que la mayor concentración de casos se mantuvo en la misma ubicación a lo largo del tiempo, a pesar de la dispersión de los casos en el territorio municipal. La persistencia del foco endémico a lo largo del tiempo plantea interrogantes sobre las estrategias de control utilizadas hasta ahora, ya que no parecen haber sido suficientes para contener la propagación del virus en esta área específica.
